# Pax6 Lengthens G1 Phase and Decreases Oscillating Cdk6 Levels in Murine Embryonic Cortical Progenitors

**DOI:** 10.3389/fncel.2018.00419

**Published:** 2018-11-15

**Authors:** Da Mi, Martine Manuel, Yu-Ting Huang, John O. Mason, David J. Price

**Affiliations:** Biomedical Sciences, The University of Edinburgh, Edinburgh, United Kingdom

**Keywords:** Pax6, cortex, Cdk6, progenitor, cell cycle

## Abstract

Pax6 is a key regulator of the rates of progenitor cell division in cerebral corticogenesis. Previous work has suggested that this action is mediated at least in part by regulation of the cell cycle gene *Cdk6*, which acts largely on the transition from G1 to S phase. We began the present study by investigating whether, in addition to *Cdk6*, other Pax6-regulated cell cycle genes are likely to be primary mediators of Pax6’s actions on cortical progenitor cell cycles. Following acute cortex-specific deletion of Pax6, *Cdk6* showed changes in expression a day earlier than any other Pax6-regulated cell cycle gene suggesting that it is the primary mediator of Pax6’s actions. We then used flow cytometry to show that progenitors lacking Pax6 have a shortened G1 phase and that their Cdk6 levels are increased in all phases. We found that Cdk6 levels oscillate during the cell cycle, increasing from G1 to M phase. We propose a model in which loss of Pax6 shortens G1 phase by raising overall Cdk6 levels, thereby shortening the time taken for Cdk6 levels to cross a threshold triggering transition to S phase.

## Introduction

Cerebral corticogenesis requires precise spatio-temporal control of the rates of progenitor cell division. Previous research on the developing mouse embryo established that the transcription factor Pax6 is a key regulator of this process (Götz et al., 1998; Warren et al., 1999; Estivill-Torrus et al., 2002; Heins et al., 2002; Haubst, 2004; Manuel et al., 2006, 2015; Quinn et al., 2007; Sansom et al., 2009; Asami et al., 2011; Georgala et al., 2011a,b; Mi et al., 2013). Pax6 is expressed by cortical radial glial progenitors, which are by far the most numerous cortical cell type present at the onset of corticogenesis at around embryonic day 12.5 (E12.5) in mouse. Previous functional studies have shown that Pax6 is an important cell autonomous repressor of radial glial cell cycle progression (Warren et al., 1999; Estivill-Torrus et al., 2002; Manuel et al., 2006; Georgala et al., 2011b; Mi et al., 2013). Loss of Pax6 shortens the overall length of cortical progenitor cell cycles and hence accelerates cell division (Mi et al., 2013). Previously, we identified a number of highly conserved cell cycle genes with actions fundamental to cell cycle progression across cell types and species whose levels of expression are affected by constitutive loss of Pax6 function (Nurse, 2000, 2002). One of these was the cell cycle gene Cdk6 (Mi et al., 2013).

Cdks drive progression through the eukaryotic cell cycle by forming complexes with cyclins (Morgan, 1997; Loog and Morgan, 2005; Hochegger et al., 2008; Coudreuse and Nurse, 2010; Uhlmann et al., 2011). In most natural systems studied so far, levels of Cdks remain relatively constant during the cell cycle whereas levels of the cyclins oscillate, causing Cdk activity to oscillate. In experiments using engineered fission yeast, oscillations in Cdk activity alone, without the numerous additional regulatory mechanisms that normally affect the cell cycle, can be sufficient to drive orderly progression through major cell cycle events (Coudreuse and Nurse, 2010). Cdk6 specifically partners a particular type of cyclin (the D-type cyclins) and primarily regulates the G1 to S phase transition (Hochegger et al., 2008). In cerebral cortical progenitors, Pax6 negatively regulates the expression of Cdk6 (Mi et al., 2013).

Here we addressed several questions. Are the effects of Pax6 on the cell cycle likely to be mediated primarily through actions on Cdk6, or does Pax6 control multiple cell cycle regulators in parallel? Do the effects of Pax6 vary between cell cycle phases? Are levels of Cdk6 normally constant throughout the cell cycle of cortical progenitors? Does Pax6 modulate Cdk6 levels equally or differentially across the phases?

To address the first of these questions, we used quantitative real time polymerase chain reaction (qRT-PCR) to test for changes in the expression of a set of cell cycle regulators following acute cortical deletion of Pax6. The set we tested contained all cell cycle regulators that were shown in previous work to have altered cortical expression levels in constitutive *Pax6*^-/-^ mutants (Mi et al., 2013). Here we studied embryos with tamoxifen-induced cortex-specific deletion of Pax6. This allowed us to focus on the effects of Pax6 loss that are more likely to be direct by minimizing the possibility of secondary changes arising as a long-term consequence of cortical deletion or Pax6 removal from non-cortical tissues. We then used flow cytometry to discover how Pax6 affects the phases of the cell cycle and the levels of Cdk6 in each. We found that Pax6 loss increases Cdk6 levels similarly in all cell cycle phases and, particularly interestingly, that Cdk6 levels oscillate with cell cycle phase. This prompted us to extend our analysis to examine the relationship between Pax6 levels and Cdk6 levels across cell cycle phases. Our results indicated that Pax6 sets the level of expression around which Cdk6 oscillates but is unlikely to be responsible for controlling the oscillation itself.

## Materials and Methods

### Mice and Tissue Preparation

All mice were bred in-house. The University of Edinburgh Animal Welfare and Ethics Board and a license from the Home Office UK issued under the UK Animals (Scientific Procedures) Act 1986 regulated all procedures. For constitutive inactivation of Pax6, we used the *Pax6*^Sey^ allele (designated as *Pax6^-^* here; (Hill et al., 1991). For conditional inactivation of Pax6, we used the *Pax6*^loxP^ allele (Simpson et al., 2009) and a green fluorescent protein (GFP) reporter allele (Sousa et al., 2009) with BAC transgenic strain *Emx1*^CreERT2^ (Kessaris et al., 2006). Dams were killed by cervical dislocation, fetuses were removed and cortices were dissected and used for RNA extraction (RNeasy Plus micro kit; Qiagen, Hilden, Germany) or dissociated with papain (20 U ml^-1^; Dissociation kit; Worthington Biochemical Corporation, Lakewood, NJ, United States). Dissociated cell suspensions diluted to 1.8 × 10^6^ ml^-1^ were fixed and permeabilised in 100% ethanol and stored at -20°C.

### qRT-PCR

cDNA was synthesized from RNA with a Superscript reverse transcriptase reaction (Thermo Fisher Scientific, Perth, United Kingdom) and qRT-PCR was done with a Quantitect SYBR Green PCR kit (Qiagen, Hilden, Germany) and a DNA Engine Opticon Continuous Fluorescence Detector (MJ Research, QC, Canada). Primer pairs are listed in Table 1. For each sample, we normalized the abundance of each transcript to the GAPDH expression level and calculated the ratios of normalized expression levels in conditional knock outs (cKOs) to littermate controls. We used three biological replicates from three different litters. For each biological replicate we ran three technical replicates.

**Table 1:** qRT-PCR primers.

Gene	Forward primer	Reverse primer
*Pax6* (exons 6–7)	TATTACGAGACTGGCTCCAT	TTGATGACACACTGGGTATG
*GFP*	CAGCCACAACGTCTATATCA	GTGTTCTGCTGGTAGTGGTC
*Cdk6*	GAGTGTCGGTTGCATCTTT	GAGTCCAATGATGTCCAAGA
*Cdca7*	GGAACGTCCATGCTTACTTG	CACAACGTCGAGAACAAGAG
*Smad2*	GCAGGAATTGAGCCACAGAG	CGGAGAGCCTGTGTCCATAC
*Smad3*	GATGACTACAGCCATTCCAT	TCACTGGTTTCTCCATCTTC
*Smad4*	TCCTGTGGCTTCCACAAGTC	ATGGTAAGTAGCTGGCTGAG
*Smad7*	GTGTTGCTGTGAATCTTACG	CCATTGGGTATCTGGAGTAA
*Cdk2*	GTGTACCCAGTACTGCCATC	TCCATGAATTTCTTGAGGTC
*Cdk4*	GAGGACATACCTGGACAAAG	AGAATGTTCTCTGGCTTCAG
*Ccnd1*	TTCATCGAACACTTCCTCTC	GAGGGTGGGTTGGAAAT
*Ccnd2*	AGTGTGCATGTTCCTAGCTT	CAGGTTCCACTTCAGCTTAC
*Sesn1*	TTGGCTGATTACCAAAGAAC	GAGGCAAGAGAGTGGTAGTG
*Mcm3*	GGACGATATAGCCAAGATCA	GAGGATTGCCTTCTTGACAT
*Mcm6*	GATTGTTGTGCCTGATGTCT	GACCAGCCTGTATGACAGAT
*Smc2*	GACCAGAACTGTAACCCTTG	AGTTCATTCTCCTTGGTCCT
*Ccdc80*	ATCTTTGGTCCTGTCAACAA	CATTCCATACTCCTTCCTCA
*Ccdc90A*	GCACAGAAAAGAGAACTTGC	CAGTGCAGACACAATGATTT


### Flow Cytometry

Aliquots of permeabilised and fixed cells suspended in fluorescence activated cell sorting (FACS) buffer were reacted with Hoechst 33342 (Thermo Fisher Scientific, Perth, United Kingdom; Cat# 1391095). Primary antibodies used were: anti-Pax6 (1:200, rabbit, Millipore, Livingston, United Kingdom; Cat# AB2237); anti-Tuj1 (1:800, mouse, Cambridge Bioscience, Cambridge, United Kingdom; Cat# MMS 435P); anti-PH3 (1:200, mouse, Cell Signaling Technology, Danvers, Massachusetts, United States; Cat# 9706); anti-Ki67 (1:500, rat, eBioscience, San Diego, CA, United States; Cat# 12-5698); anti-Cdk6 (1:500, rabbit, Santa Cruz Biotechnology, Dallas, TX, United States; Cat# SC-177). We used secondary-only and fluorescence-minus-one controls to set thresholds for primary antibody-specific staining. Cells were analyzed using an LSRII flow cytometer and FlowJo V10 software (BD Biosciences, Franklin Lakes, NJ, United States). In all of our experiments, we used the same gate for forward scatter for both wild-type and mutant progenitor cells. Since forward scatter values in flow cytometry are directly linked to cell size, this indicates that the wild-type and mutant cells were in the same size range.

### Immunohistochemistry for BrdU and IdU

Pregnant females were injected intra-peritoneally with 200 μl of 100 μg ml^-1^ IdU and then 1.5 h later with the same dose of BrdU. They were sacrificed after 30 min and histological sections were cut through the cortex. Primary antibodies used were anti-BrdU/IddU (which recognizes both BrdU and IddU) (1:100, mouse, Becton Dickinson, Swindon, United Kingdom; clone B44) and anti-BrdU (1:100, rat, Abcam, Cambridge, United Kingdom; clone BU1/75). Nuclei were counterstained with TO-PRO-3 iodide (Molecular Probes). Analysis was as described in (Martynoga et al., 2005).

## Results

### Changes in the Expression of Cell Cycle Regulators Following Acute Cortical Deletion of Pax6

**FIGURE 1:**
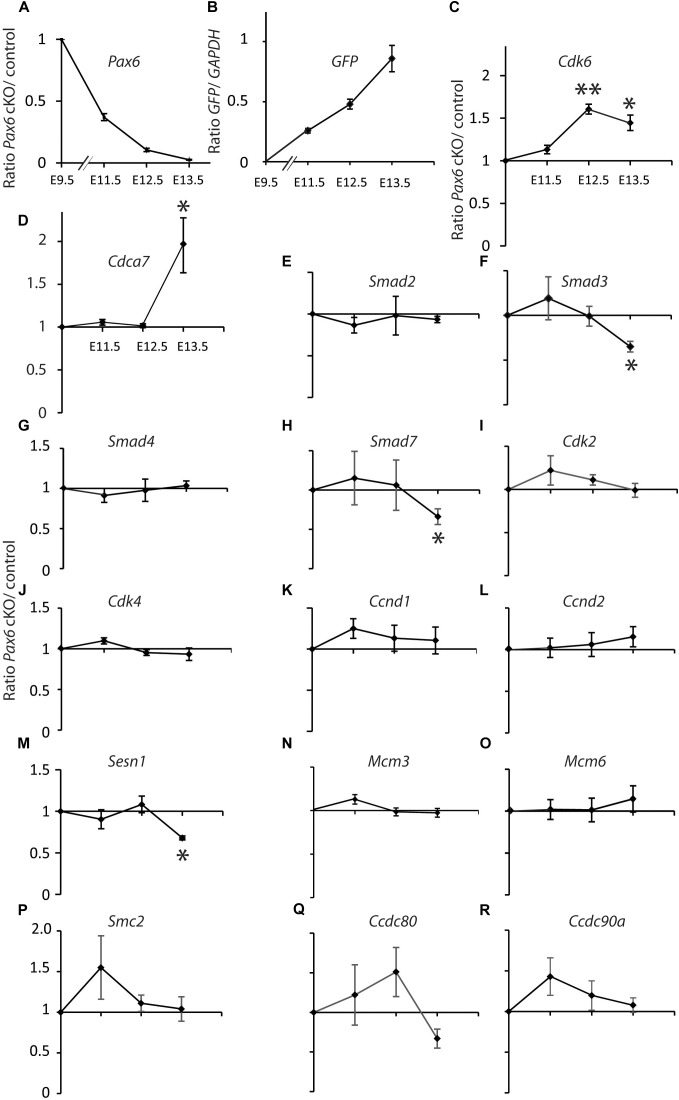
Quantitative RT-PCR analysis of the effects of tamoxifen-induced cortex-specific Pax6 deletion on the expression of cell cycle genes. **(A)** Tamoxifen given at E9.5 resulted in *Pax6* mRNA loss, with a ∼60% reduction by E11.5 and a ∼100% reduction by E13.5. **(B)** This loss coincided with the production of increasing levels of *GFP* reporter gene mRNA. **(C–R)** Changes in the expression of 16 cell cycle genes. In all cases, the levels of cortical expression of each gene were measured relative to levels of cortical expression of *GAPDH* in the same embryo. In panels **(A)** and **(C–R)**, ratios of expression in conditional knock-outs (cKO) over littermate controls were combined to give mean ± SEM at each age. All data are from three biological replicates at each age; ^∗^*p* < 0.05; ^∗∗^*p* < 0.01; Student’s *t*-test comparison of cKOs with paired controls at each age.

We gave tamoxifen to experimental *Pax6*^fl/fl^;*Emx1*^CreER^ (designated cKO) and littermate control *Pax6*^fl/+^;*Emx1*^CreER^ embryos at E9.5. All embryos contained a floxed-stop *GFP* reporter allele (Sousa et al., 2009). We used qRT-PCR to measure levels of mRNA in cortical samples relative to levels of *GAPDH*. For all genes except *GFP* we then calculated ratios between the relative mRNA levels in cKO and control littermates at E11.5, 12.5, and 13.5 (Figure 1).

*Pax6* levels decayed exponentially and *GFP* levels rose steadily over the 4 days following tamoxifen administration (Figures 1A,B). *Pax6* levels had declined to ∼40% of normal by E11.5, the earliest post-tamoxifen time-point examined. *Cdk6* was the only cell cycle gene studied that showed a significant change (upregulation) in gene expression at E12.5 and this was sustained at E13.5 (Figure 1C). *Cdca7* showed a significant upregulation but only later, at E13.5 (Figure 1D). *Smad3*, *Smad7*, and *Sesn1* showed significant downregulation but only at E13.5 (Figures 1F,H,M). These results suggest that no other Pax6-regulated cell cycle gene rivals *Cdk6* as the top candidate among possible mediators of Pax6’s actions on cortical progenitor cell cycle lengths. Whereas genes such as *Cdca7*, *Smad3*, *Smad7*, and *Sesn1* are almost certainly involved, it is possible that they alter their expression as a secondary consequence of changes in *Cdk6* expression.

### G1 Is Abnormally Short in *Pax6*^-/-^ Mutants

**FIGURE 2:**
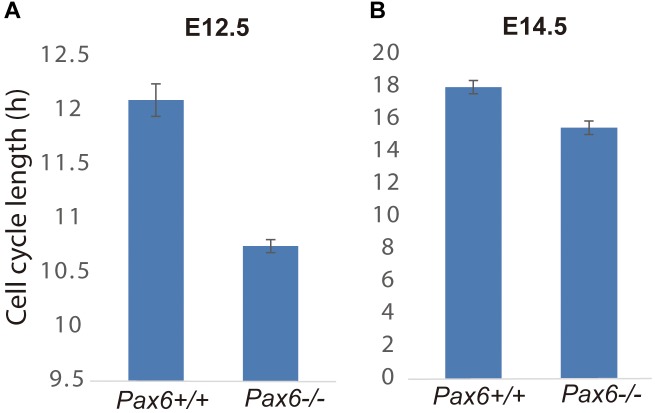
Cell cycle lengths are shortened in *Pax6*^-/-^ progenitors. Lengths of the cell cycles of progenitors in *Pax6*^+/+^ and *Pax6*^-/-^ E12.5 and E14.5 cortex measured using double labeling with IdU and BrdU [*n* = 3 embryos of each genotype from three different litters for each age; mean ± SEMs are shown; in panel **(A)**, *p* = 0.0099, Student’s *t*-test; in panel **(B)**, *p* = 0.0004, Student’s *t*-test].

Given that Cdk6’s main action during the eukaryotic cell cycle is to promote the G1 to S phase transition (Hochegger et al., 2008), we hypothesized that elevation of Cdk6 in *Pax6*^-/-^ mutants might shorten the cell cycle of cortical progenitors by shortening primarily G1 phase. We first estimated the overall lengths of the cell cycles (Tc) of progenitors in *Pax6*^+/+^ and *Pax6*^-/-^ E12.5 and E14.5 cortex using double labeling with iododeoxyuridine (IdU) and bromodeoxyuridine (BrdU) (Martynoga et al., 2005). At E12.5, Tc was ∼10% shorter in *Pax6*^-/-^ cortex than in *Pax6*^+/+^ cortex (*n* = 3 embryos of each genotype from three different litters; mean Tc ± SEM = 12.1 ± 0.15 h in *Pax6*^+/+^ and 10.75 ± 0.06 h in *Pax6*^-/-^; *p* = 0.0099, Student’s *t*-test). At E14.5, Tc was ∼15% shorter in *Pax6*^-/-^ cortex than in *Pax6*^+/+^ cortex (*n* = 3 embryos of each genotype from three different litters; mean Tc ± SEM = 18.0 ± 0.41 h in *Pax6*^+/+^ and 15.5 ± 0.42 h in *Pax6*^-/-^; *p* = 0.0004, Student’s *t*-test) (Figure 2).

**FIGURE 3:**
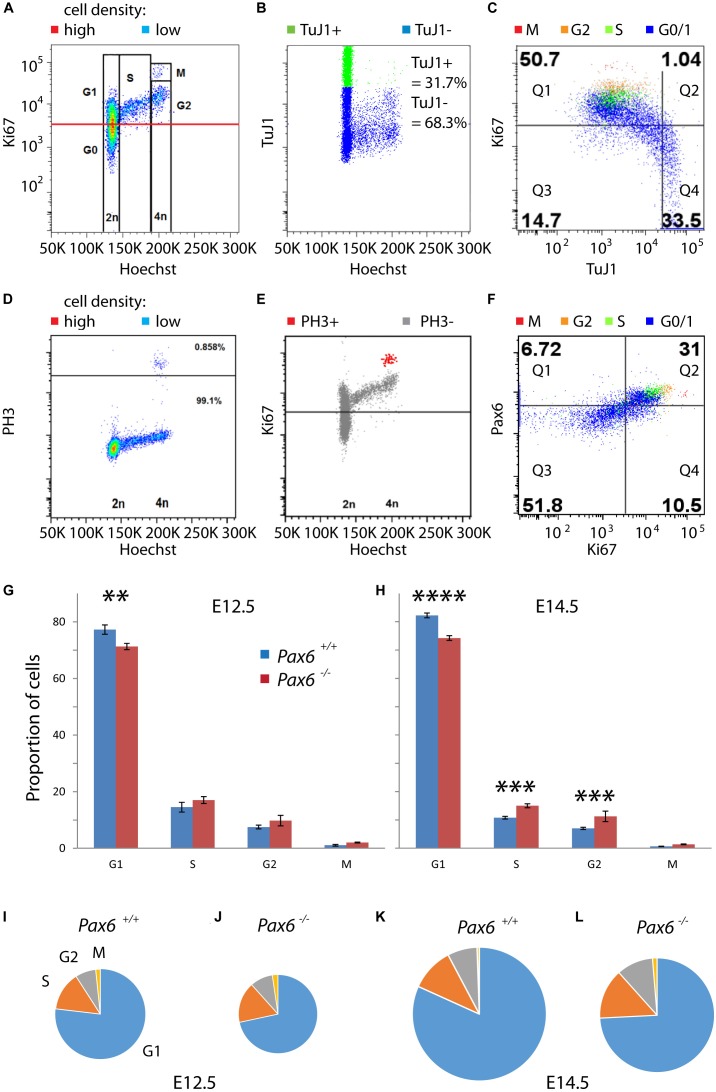
Flow cytometric analysis of cell cycle phases of cortical progenitors in *Pax6*^+/+^ and *Pax6*^-/-^ embryos at E12.5 and E14.5. **(A–F)** Representative examples of flow cytometry showing how cells were assigned to specific stages of the cell cycle. Cells were labeled with Hoechst 33342 and antibodies for Ki67, class III beta-tubulin (TuJ1), phospho-Histone H3 (PH3) and Pax6. **(A)** Fluorescence intensity of Ki67 against Hoechst, shown as a heat-map with red areas containing most cells and blue areas fewest: Cells were classified as G1 if they had 2n DNA content and were positive for Ki67 (intensity values above the red line), which is a marker of proliferating cells. **(B)** Fluorescence intensity of TuJ1 against Hoechst: TuJ1 was used as a marker of differentiating neurons. **(C)** Fluorescence intensity of Ki67 against TuJ1, with different colors indicating cell cycle phase of each cell: In this sample, 98.0% (50.7/51.74) of Ki67+ cells were TuJ1– whereas 69.3% (33.5/48.3) of Ki67– cells were Tuj1+. **(D)** Fluorescence intensity of PH3 against Hoechst, plotted using the same method as in panel **(A)**: Cells in M phase were identified by their expression of PH3 (0.858%, above black line). **(E)** Fluorescence intensity of Ki67 against Hoechst: M phase cells expressed PH3 and contained the highest levels of Ki67. **(F)** Fluorescence intensity of Pax6 against Ki67, plotted using the same method as in panel **(C)**: Very few Ki67– cells expressed Pax6 (11.5% [6.72/58.52] in this sample) whereas most Ki67+ cells co-expressed Pax6 (74.7% [31/40.5] in this sample). **(G,H)** Proportions of proliferating cells (i.e., G0 cells excluded) in each phase of the cell cycle in *Pax6*^+/+^ and *Pax6*^-/-^ embryos at E12.5 and E14.5. Values are mean ± SEM (*n* = 4 embryos in all cases). ANOVA showed a significant effect of genotype at both ages (α = 0.05). Significant effects of genotype on each phase at each age are marked (Sidak’s multiple comparisons tests: ^∗∗^*p* = 0.0029; ^∗∗∗^*p* = 0.0001; ^∗∗∗∗^*p* < 0.0001). **(I–L)** Charts, drawn to scale, summarizing the relative lengths of the cell cycle phases across ages and genotypes.

We then used flow cytometry to quantify the relative lengths of G1, S, G2, and M phases in cortical cells from E12.5 and E14.5 *Pax6*^+/+^ and *Pax6*^-/-^ embryos (Figure 3). A combination of Hoechst and antibody staining identified cells in each of the phases (Figures 3A–F). Cells were labeled with Hoechst 33342 to measure DNA content and antibodies for Ki67 (a marker of proliferating cells), class III beta-tubulin (TuJ1; a marker of postmitotic neurons), phospho-Histone H3 (PH3; a marker of M phase), and Pax6. Cells were classified as G1 if they had 2n DNA content and were positive for Ki67 (Figure 3A). As expected, almost all of these cells (∼98%) were negative for TuJ1 (Figure 3C). Combining data from multiple embryos (four per genotype per age) showed significant reductions by ∼8–10% in the proportions of cells in G1 at both ages in *Pax6*^-/-^ embryos (Figures 3G,H). For phases other than G1, the only significant differences were in S and G2 at E14.5 (Figures 3G,H), where mean values were higher in mutants in both cases.

We estimated the lengths of each phase in hours under different conditions by multiplying the proportion of cells in that phase by the corresponding Tc derived from calculations above. These estimations gave values for G1 of 9.3 h in *Pax6*^+/+^ cortex and 7.7 h in *Pax6*^-/-^ cortex at E12.5. At E14.5, values for G1 were 14.8 h in *Pax6*^+/+^ cortex and 11.5 h in *Pax6*^-/-^ cortex. At E12.5, S phase was 1.7 h in *Pax6*^+/+^ cortex versus 1.8 h in *Pax6*^-/-^ cortex, G2 phase was 0.9 h in *Pax6*^+/+^ cortex versus 1.0 h in *Pax6*^-/-^ cortex and M phase was 0.2 h in *Pax6*^+/+^ cortex versus 0.25 h in *Pax6*^-/-^ cortex. At E14.5, S phase was 1.9 h in *Pax6*^+/+^ cortex versus 2.2 h in *Pax6*^-/-^ cortex, G2 phase was 1.3 h in *Pax6*^+/+^ cortex versus 1.6 h in *Pax6*^-/-^ cortex, and M phase was 0.1 h in *Pax6*^+/+^ cortex versus 0.2 h in *Pax6*^-/-^ cortex. These estimations are summarized graphically in Figures 3I–L. These data suggest that in the absence of Pax6 G1 phase is shortened by ∼2–3 h while other phases are similar to or ∼15–20 min longer than normal.

### Cdk6 Levels Are Elevated During All Phases of the Cell Cycle in *Pax6*^-/-^ Cortical Progenitors

**FIGURE 4:**
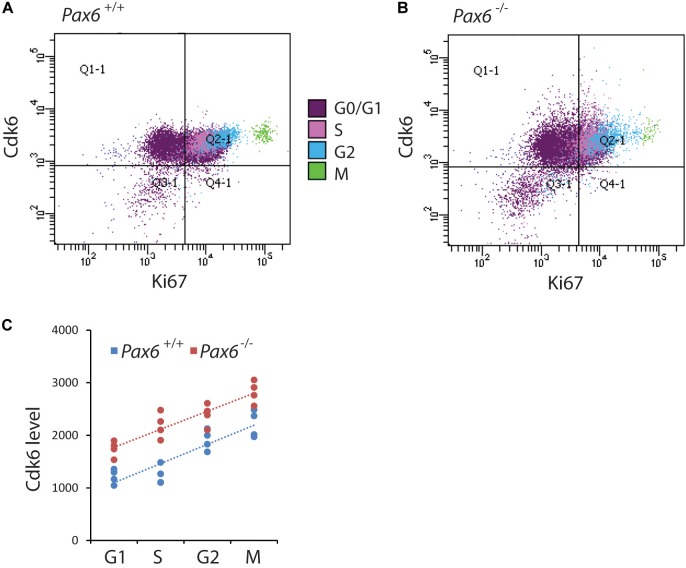
Flow cytometric analysis of Cdk6 levels at different phases of the cell cycle in *Pax6*^+/+^ and *Pax6*^-/-^ cortex at E12.5. **(A,B)** Fluorescence intensity of Cdk6 against Ki67: Samples from a *Pax6*^+/+^ and a *Pax6*^-/-^ embryo with cells classified according to their cell cycle phase by methods illustrated in Figure 3. G1 cells were distinguished from G0 cells by positivity for Ki67 in quadrants Q2-1 and Q4-1 (cells in quadrants 1-1 and 3-1 were negative for Ki67). **(C)** Relative levels of Cdk6 in the cortex of four *Pax6*^+/+^ and four *Pax6*^-/-^ embryos at each phase of the cell cycle. Each data point is the average level in the cortex of one embryo. Regression analysis of levels of Cdk6 against cells cycle phase gave *r*^2^ values of 0.78 for *Pax6*^+/+^ data and 0.81 for *Pax6*^-/-^ data. ANOVA showed a significant effect of genotype (α = 0.05) and Sidak’s multiple comparisons tests showed significantly higher levels of Cdk6 in *Pax6*^-/-^ cortex in all phases (G1, *p* = 0.0075; S, *p* < 0.0001; G2, *p* = 0.0167; M, *p* = 0.0019).

In most systems studied to date, levels of Cdks remain relatively constant during the cell cycle (Hochegger et al., 2008). It was not known whether this is also the case for cortical progenitors and whether the absence of Pax6 affects Cdk6 levels in all phases of the cortical progenitor cell cycle. We used flow cytometry to identify cells in each cell cycle phase (Figures 3A–F) and measure their Cdk6 levels. We found significantly elevated levels of Cdk6 in all cell cycle phases of *Pax6*^-/-^ progenitors (Figure 4C). In addition, Cdk6 levels varied across the phases, rising by ∼1.5–2-fold from G1 to M both in *Pax6*^+/+^ and *Pax6*^-/-^ progenitors (Figure 4C).

### Pax6 Levels Oscillate During the Cell Cycle in Cortical Progenitors

**FIGURE 5:**
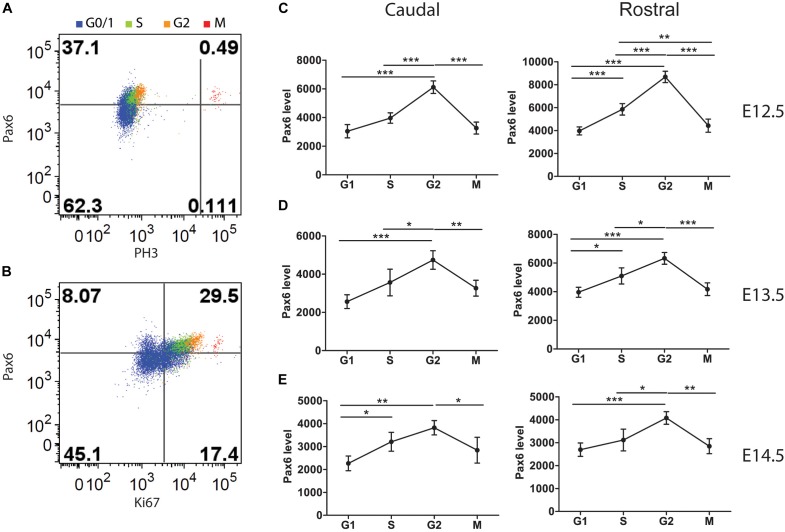
Flow cytometric analysis of Pax6 levels at different phases of the cell cycle in *Pax6*^+/+^ cortex at E12.5, E13.5, and E14.5. Cells classified according to their cell cycle phase by methods illustrated in Figure 1. **(A)** Example showing how M phase cells were identified by their expression of PH3. **(B)** Example showing how G1 cells were distinguished from G0 cells by positivity for Ki67. **(C–E)** Average relative levels (± SEM) of Pax6 in proliferating cortical cells (G0 cells excluded) in each cell cycle phase in rostral and caudal cortex (*n* = 4 embryos at each age). ANOVAs at each age showed significant effects of cell cycle phase (α = 0.05). Sidak’s multiple comparisons tests showed significant differences between phases, as marked (^∗^*p* < 0.05; ^∗∗^*p* < 0.001; ^∗∗∗^*p* < 0.0001).

A parsimonious explanation for the fact that Cdk6 levels oscillate not only in *Pax6*^+/+^ cortex but also in *Pax6*^-/-^ cortex is that Pax6 influences the average level of Cdk6 expression rather than being directly responsible for the oscillations themselves, which would be caused by a different mechanism. This would explain why Cdk6 levels still oscillate in the absence of Pax6. Nonetheless, a more complex scenario was conceivable. In normal cortex, Pax6 levels might oscillate during the cell cycle in such a way as to drive changes in Cdk6 levels; in *Pax6*^-/-^ cortex, some other unknown mechanism might take over Pax6’s role. We addressed whether this second possibility might be feasible by measuring Pax6 levels in each phase of the cell cycle in normal embryos using flow cytometry (Figure 5).

We identified cells in each phase using methods illustrated in Figures 3, 5A,B. We found that Pax6 levels varied significantly with cell cycle phase at all ages studied (Figures 5C–E). In most phases at E12.5 and E13.5 (the one exception being M phase at E13.5), Pax6 levels were significantly higher in rostral than in caudal cortex (*p* < 0.01 in all phases at both ages, Sidak’s multiple comparisons tests following ANOVA, α = 0.05). This was only the case in G1 at E14.5 (*p* < 0.01). These findings on rostral versus caudal differences are in excellent agreement with previous work showing a rostral [high] to caudal [low] gradient of Pax6 expression at E12.5 and E13.5 that flattens by E14.5 (Mi et al., 2013).

Pax6 levels changed by ∼1.5–2-fold from trough to peak during the cell cycle (Figures 5C–E). The oscillations in Pax6 levels were out of phase with the oscillations in Cdk6 levels shown in Figure 4. Levels rose to a peak in G2 and dropped in M, before Cdk6 levels fell. The rise of Pax6 from G1 to G2 paralleled that of Cdk6 through these three phases.

## Discussion

**FIGURE 6:**
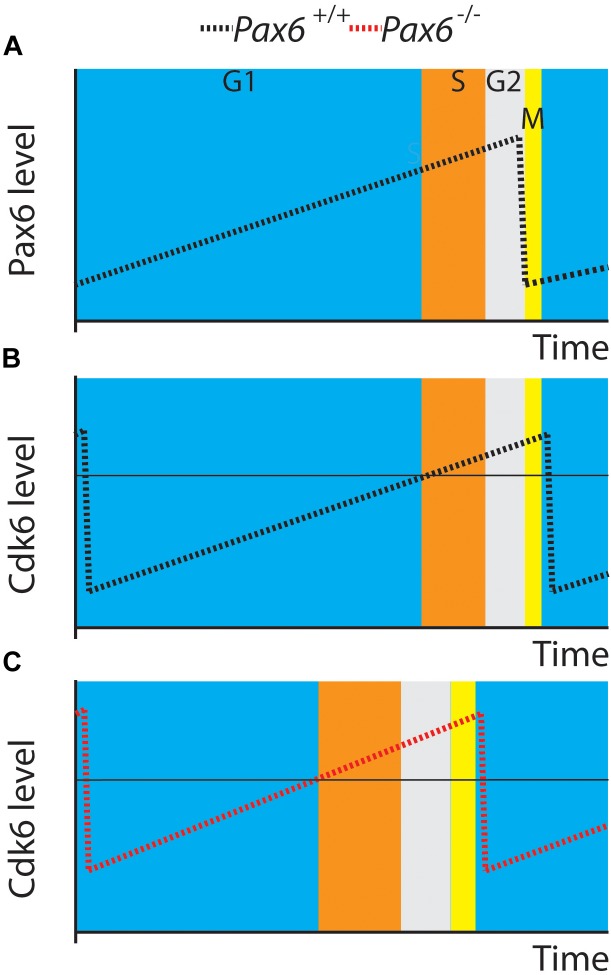
A model. **(A,B)** Pax6 and Cdk6 oscillations are slightly out of phase. **(C)** A general elevation of Cdk6 activity throughout the cell cycle might allow levels critical for switching from G1 to S (horizontal line in **B,C**) to be achieved more rapidly than normal.

Our new results support a central role for Cdk6 in Pax6’s regulation of cortical progenitor cell cycles. Figure 6 shows a model illustrating how elevated Cdk6 levels across the cell cycle could shorten G1 phase. We propose that the time of G1 and the G1 to S phase transition is determined by the time it takes Cdk6 activity to rise above a threshold; our model proposes that the threshold is similar in both *Pax6*^+/+^ and *Pax6*^-/-^ progenitors (Figures 6B,C). Oscillation of Cdk6 activity at abnormally high levels would shorten the length of G1 since it would take less time to achieve the threshold required to exit G1. We also found evidence that other cell cycle phases were slightly longer than normal in *Pax6*^-/-^ progenitors, particularly at the later age studied, E14.5. This is reflected in the way Figure 6 is drawn. These changes were not enough to compensate for the shortening of G1 and, overall, cell cycle lengths were reduced. We do not know how these other phases might be affected. At least one possibility is that they are lengthened as a consequence of accelerated progression through G1 phase rather than a direct action of Pax6 on regulators of these phases. Another possibility is that delayed changes in the expression of cell cycle genes other than *Cdk6*, such as those observed here (Figure 1: *Cdca7*, *Smad3*, *Smad7*, or *Sesn1*) directly affect the lengths of these phases.

This proposal has similarity to the mechanism proposed to regulate an engineered minimal control network in fission yeast (Coudreuse and Nurse, 2010). This control network is based on oscillating levels of CDK activity, which drive cell cycle progression as they cross thresholds triggering transition between cell cycle phases including G1 to S. As in the fission yeast model, it is possible that the oscillations in Cdk6 in cortical progenitors result from phase-dependent changes in Cdk6 synthesis and degradation.

An alternative possibility is that cyclical changes in Pax6 levels might cause the oscillations in Cdk6 levels, given that Pax6 can directly repress Cdk6 expression (Mi et al., 2013). The oscillations in Pax6 were slightly out of phase with those in Cdk6 (Figures 6A,B) but it is very unlikely that fluctuations in Pax6 protein levels alone cause the oscillations in Cdk6 protein levels. Additional factors would be required to explain the very different rates of change of Pax6 during its rising phase and Cdk6 during its falling phase and *vice versa*. An additional complexity would be explaining the dynamics of this scenario. Changes in Pax6 protein levels would need a certain time to influence transcription of the *Cdk6* gene and the mRNA would then need to be translated to alter Cdk6 protein levels. Although we do not exclude the possibility that fluctuations in Pax6 levels might contribute to the temporal pattern of change of Cdk6 levels, the simplest suggestion is that Pax6 reduces Cdk6 levels overall and that the oscillations in Cdk6 levels during the cell cycle are driven by other mechanisms. Our previous work showed that Pax6 overexpression significantly reduced Cdk6 levels (Mi et al., 2013), and in the future it would be interesting to test how Cdk6 levels oscillate during the cell cycle in these mutants and how cell cycle phases change.

It is likely that post-translational processes are involved in the relatively rapid cyclical changes in Cdk6 and Pax6 levels identified here. Cortical progenitor cells might generate excess amounts of their mRNAs, which might be translated at levels that vary as the cell progresses through the cell cycle. A similar mechanism, termed “translation on demand,” was suggested in a recently published review paper (Liu et al., 2016). It suggests that quick generation of proteins in response to signals could be achieved purely by regulating the translation rate of pre-existing mRNA (Le Roch et al., 2004; Lee et al., 2011; Lackner et al., 2012; Eichelbaum and Krijgsveld, 2014; Jovanovic et al., 2015). Some studies suggested that transcription factors are more likely to be subject to translation on demand regulation, as some rapid cell state transitions require fast synthesis and reactions of transcription factors to modulate their downstream gene networks (Lee et al., 2013; Jovanovic et al., 2015; Liu et al., 2016). Rapid degradation via ubiquitination might also contribute to rapid adjustments of Pax6 and Cdk6 protein levels during the cell cycle. In eye development, for example, a post-translational ubiquitin-mediated proteasomal mechanism degrades Pax6 protein (Pfirrmann et al., 2016). It is possible that Pax6 regulates the overall levels of Cdk6 not only by direct repression of the *Cdk6* gene itself (Mi et al., 2013) but also by affecting the post-translational mechanisms that determine Cdk6 protein production and stability.

A Pax6-loss-induced change in the length of G1 phase is likely to have consequences for the subsequent development of progenitors and the neurons that they generate. Previous studies have shown that cell cycle length has a direct impact on a cell’s mode of division, i.e., whether it is neurogenic, producing neurons, or proliferative, producing new progenitor(s). G1 length is increased in neurogenic progenitors compared with proliferative progenitors (Caviness et al., 2003; Lukaszewicz et al., 2005; Dehay and Kennedy, 2007; Salomoni and Calegari, 2010). This is in line with the finding that basal progenitors, which mostly generate neurons through neurogenic division, have a longer G1 phase than apical progenitors (Calegari et al., 2005; Salomoni and Calegari, 2010; Arai et al., 2011). The functional importance of G1 length was demonstrated by manipulating it. An increase in G1 length leads to neurogenic division and premature neurogenesis (Calegari, 2003; Calegari et al., 2005). Shortening G1 increases proliferative divisions, leading to an expansion of the progenitor pool, and affects laminar phenotypes later in development (Lange et al., 2009; Pilaz et al., 2009). This might be one mechanism by which Pax6 affects the later stages of cortical formation despite being downregulated in postmitotic neurons.

## Author Contributions

DM, MM, and Y-TH performed the experiments. DP and JM helped to design the experiments, obtained the funding, and supervised the work. DP wrote the manuscript.

## Conflict of Interest Statement

The authors declare that the research was conducted in the absence of any commercial or financial relationships that could be construed as a potential conflict of interest.

## References

[B1] Arai Y. Pulvers J. N. Haffner C. Schilling B. Nüsslein I. Calegari F. (2011). Neural stem and progenitor cells shorten S-phase on commitment to neuron production. *Nat. Commun.* 2:54. 10.1038/ncomms1155 21224845 PMC3105305

[B2] Asami M. Pilz G. A. Ninkovic J. Godinho L. Schroeder T. Huttner W. B. (2011). The role of Pax6 in regulating the orientation and mode of cell division of progenitors in the mouse cerebral cortex. *Development* 138 5067–5078. 10.1242/dev.074591 22031545

[B3] Calegari F. (2003). An inhibition of cyclin-dependent kinases that lengthens, but does not arrest, neuroepithelial cell cycle induces premature neurogenesis. *J. Cell Sci.* 116(Pt 24), 4947–4955. 10.1242/jcs.00825 14625388

[B4] Calegari F. Haubensak W. Haffner C. Huttner W. B. (2005). Selective lengthening of the cell cycle in the neurogenic subpopulation of neural progenitor cells during mouse brain development. *J. Neurosci.* 25 6533–6538. 10.1523/JNEUROSCI.0778-05.2005 16014714 PMC6725437

[B5] Caviness V. S. Goto T. Tarui T. Takahashi T. Bhide P. G. Nowakowski R. S. (2003). Cell output, cell cycle duration and neuronal specification: a model of integrated mechanisms of the neocortical proliferative process. *Cereb. Cortex* 13 592–598. 10.1093/cercor/13.6.592 12764033

[B6] Coudreuse D. Nurse P. (2010). Driving the cell cycle with a minimal CDK control network. *Nature* 13 592–598. 10.1038/nature09543 21179163

[B7] Dehay C. Kennedy H. (2007). Cell-cycle control and cortical development. *Nat. Rev. Neurosci.* 8 438–450. 10.1038/nrn2097 17514197

[B8] Eichelbaum K. Krijgsveld J. (2014). Rapid temporal dynamics of transcription, protein synthesis, and secretion during macrophage activation. *Mol. Cell. Proteomics* 13 792–810. 10.1074/mcp.M113.030916 24396086 PMC3945909

[B9] Estivill-Torrus G. Pearson H. van Heyningen V. Price D. J. Rashbass P. (2002). Pax6 is required to regulate the cell cycle and the rate of progression from symmetrical to asymmetrical division in mammalian cortical progenitors. *Development* 129 455–466. 11807037 10.1242/dev.129.2.455

[B10] Georgala P. A. Carr C. B. Price D. J. (2011a). The role of Pax6 in forebrain development. *Dev. Neurobiol.* 71 690–709. 10.1002/dneu.20895 21538923

[B11] Georgala P. A. Manuel M. Price D. J. (2011b). The generation of superficial cortical layers is regulated by levels of the transcription factor Pax6. *Cereb. Cortex* 21 81–94. 10.1093/cercor/bhq061 20413449 PMC3000564

[B12] Götz M. Stoykova A. Gruss P. (1998). Pax6 controls radial glia differentiation in the cerebral cortex. *Neuron* 21 1031–1044. 10.1016/S0896-6273(00)80621-2 9856459

[B13] Haubst N. (2004). Molecular dissection of Pax6 function: the specific roles of the paired domain and homeodomain in brain development. *Development* 131 6131–6140. 10.1242/dev.01524 15548580

[B14] Heins N. Malatesta P. Cecconi F. Nakafuku M. Tucker K. L. Hack M. A. (2002). Glial cells generate neurons: the role of the transcription factor Pax6. *Nat. Neurosci.* 5 308–315. 10.1038/nn828 11896398

[B15] Hill R. E. Favor J. Hogan B. L. Ton C. C. Saunders G. F. Hanson I. M. (1991). Mouse Small eye results from mutations in a paired-like homeobox-containing gene. *Nature* 354 522–525. 10.1038/354522a0 1684639

[B16] Hochegger H. Takeda S. Hunt T. (2008). Cyclin-dependent kinases and cell-cycle transitions: does one fit all? *Nat. Rev. Mol. Cell Biol.* 9 910–916. 10.1038/nrm2510 18813291

[B17] Jovanovic M. Rooney M. S. Mertins P. Przybylski D. Chevrier N. Satija R. (2015). Dynamic profiling of the protein life cycle in response to pathogens. *Science* 347:1259038. 10.1126/science.1259038 25745177 PMC4506746

[B18] Kessaris N. Fogarty M. Iannarelli P. Grist M. Wegner M. Richardson W. D. (2006). Competing waves of oligodendrocytes in the forebrain and postnatal elimination of an embryonic lineage. *Nat. Neurosci.* 9 173–179. 10.1038/nn1620 16388308 PMC6328015

[B19] Lackner D. H. Schmidt M. W. Wu S. Wolf D. A. Bähler J. (2012). Regulation of transcriptome, translation, and proteome in response to environmental stress in fission yeast. *Genome Biol.* 13:R25. 10.1186/gb-2012-13-4-r25 22512868 PMC3446299

[B20] Lange C. Huttner W. B. Calegari F. (2009). Cdk4/CyclinD1 overexpression in neural stem cells shortens G1, delays neurogenesis, and promotes the generation and expansion of basal progenitors. *Cell Stem Cell.* 5 320–331. 10.1016/j.stem.2009.05.026 19733543

[B21] Le Roch K. G. Johnson J. R. Florens L. Zhou Y. Santrosyan A. Grainger M. (2004). Global analysis of transcript and protein levels across the *Plasmodium falciparum* life cycle. *Genome Res.* 14 2308–2318. 10.1101/gr.2523904 15520293 PMC525690

[B22] Lee M. T. Bonneau A. R. Takacs C. M. Bazzini A. A. Divito K. R. Fleming E. S. (2013). Nanog, Pou5f1 and SoxB1 activate zygotic gene expression during the maternal-to-zygotic transition. *Nature* 503 360–364. 10.1038/nature12632 24056933 PMC3925760

[B23] Lee M. V. Topper S. E. Hubler S. L. Hose J. Wenger C. D. Coon J. J. (2011). A dynamic model of proteome changes reveals new roles for transcript alteration in yeast. *Mol. Syst. Biol.* 7:514. 10.1038/msb.2011.48 21772262 PMC3159980

[B24] Liu Y. Beyer A. Aebersold R. (2016). On the dependency of cellular protein levels on mRNA abundance. *Cell* 165 535–550. 10.1016/j.cell.2016.03.014 27104977

[B25] Loog M. Morgan D. O. (2005). Cyclin specificity in the phosphorylation of cyclin-dependent kinase substrates. *Nature* 434 104–108. 10.1038/nature03329 15744308

[B26] Lukaszewicz A. Savatier P. Cortay V. Giroud P. Huissoud C. Berland M. (2005). G1 phase regulation, area-specific cell cycle control, and cytoarchitectonics in the primate cortex. *Neuron* 47 353–364. 10.1016/j.neuron.2005.06.032 16055060 PMC1890568

[B27] Manuel M. Georgala P. A. Carr C. B. Chanas S. Kleinjan D. A. Martynoga B. (2006). Controlled overexpression of Pax6 in vivo negatively autoregulates the Pax6 locus, causing cell-autonomous defects of late cortical progenitor proliferation with little effect on cortical arealization. *Development* 134 545–555. 10.1242/dev.02764 17202185 PMC2386558

[B28] Manuel M. N. Mi D. Mason J. O. Price D. J. (2015). Regulation of cerebral cortical neurogenesis by the Pax6 transcription factor. *Front. Cell. Neurosci.* 9:70 10.3389/fncel.2015.00070 PMC4354436 25805971

[B29] Martynoga B. Morrison H. Price D. J. Mason J. O. (2005). Foxg1 is required for specification of ventral telencephalon and region-specific regulation of dorsal telencephalic precursor proliferation and apoptosis. *Dev. Biol.* 283 113–127. 10.1016/j.ydbio.2005.04.005 15893304

[B30] Mi D. Carr C. B. Georgala P. A. Huang Y. T. Manuel M. N. Jeanes E. (2013). Pax6 Exerts regional control of cortical progenitor proliferation via direct repression of Cdk6 and Hypophosphorylation of pRb. *Neuron* 78 269–284. 10.1016/j.neuron.2013.02.012 23622063 PMC3898967

[B31] Morgan D. O. (1997). CYCLIN-DEPENDENT KINASES: engines, clocks, and microprocessors. *Annu. Rev. Cell Dev. Biol.* 13 261–291. 10.1146/annurev.cellbio.13.1.261 9442875

[B32] Nurse P. (2000). A long twentieth century of the cell cycle and beyond. *Cell* 100 71–78. 10.1016/S0092-8674(00)81684-0 10647932

[B33] Nurse P. M. (2002). Cyclin dependent kinases and cell cycle control. *Chembiochem* 3 596–603. 10.1002/1439-7633(20020703)3:7<596::AID-CBIC596>3.0.CO;2-U 12324993

[B34] Pfirrmann T. Jandt E. Ranft S. Lokapally A. Neuhaus H. Perron M. (2016). Hedgehog-dependent E3-ligase Midline1 regulates ubiquitin-mediated proteasomal degradation of Pax6 during visual system development. *Proc. Natl. Acad. Sci. U.S.A.* 113 10103–10108. 10.1073/pnas.1600770113 27555585 PMC5018744

[B35] Pilaz L.-J. Patti D. Marcy G. Ollier E. Pfister S. Douglas R. J. (2009). Forced G1-phase reduction alters mode of division, neuron number, and laminar phenotype in the cerebral cortex. *Proc. Natl. Acad. Sci. U.S.A.* 106 21924–21929. 10.1073/pnas.0909894106 19959663 PMC2788480

[B36] Quinn J. C. Molinek M. Martynoga B. S. Zaki P. A. Faedo A. Bulfone A. (2007). Pax6 controls cerebral cortical cell number by regulating exit from the cell cycle and specifies cortical cell identity by a cell autonomous mechanism. *Dev. Biol.* 302 50–65. 10.1016/j.ydbio.2006.08.035 16979618 PMC2384163

[B37] Salomoni P. Calegari F. (2010). Cell cycle control of mammalian neural stem cells: putting a speed limit on G1. *Trends Cell Biol.* 14 2308–2318. 10.1016/j.tcb.2010.01.006 20153966

[B38] Sansom S. N. Griffiths D. S. Faedo A. Kleinjan D. J. Ruan Y. Smith J. (2009). The level of the transcription factor Pax6 is essential for controlling the balance between neural stem cell self-renewal and neurogenesis. *PLoS Genet.* 5:e1000511. 10.1371/journal.pgen.1000511 19521500 PMC2686252

[B39] Simpson T. I. Pratt T. Mason J. O. Price D. J. (2009). Normal ventral telencephalic expression of Pax6 is required for normal development of thalamocortical axons in embryonic mice. *Neural Dev.* 4:19. 10.1186/1749-8104-4-19 19500363 PMC2699344

[B40] Sousa V. H. Miyoshi G. Hjerling-Leffler J. Karayannis T. Fishell G. (2009). Characterization of Nkx6-2-derived neocortical interneuron lineages. *Cereb. Cortex* 19(Suppl. 1), i1–i10. 10.1093/cercor/bhp038 19363146 PMC2693535

[B41] Uhlmann F. Bouchoux C. Lopez-Aviles S. (2011). A quantitative model for cyclin-dependent kinase control of the cell cycle: revisited. *Philos. Trans. R. Soc. L B Biol. Sci.* 366 3572–3583. 10.1098/rstb.2011.0082 22084384 PMC3203462

[B42] Warren N. Caric D. Pratt T. Clausen J. A. Asavaritikrai P. Mason J. O. (1999). The transcription factor, Pax6, is required for cell proliferation and differentiation in the developing cerebral cortex. *Cereb. Cortex* 9 627–635. 10.1093/cercor/9.6.627 10498281

